# Molecular and Kinetic Properties of Two Acetylcholinesterases from the Western Honey Bee, *Apis mellifera*

**DOI:** 10.1371/journal.pone.0048838

**Published:** 2012-11-07

**Authors:** Young Ho Kim, Deok Jea Cha, Je Won Jung, Hyung Wook Kwon, Si Hyeock Lee

**Affiliations:** 1 Research Institute for Agriculture and Life Sciences, Seoul National University, Seoul, Korea; 2 Department of Agricultural Biotechnology, Seoul National University, Seoul, Korea; 3 WCU Biomodulation Major, Department of Agricultural Biotechnology, Seoul National University, Seoul, Korea; University of Crete, Greece

## Abstract

We investigated the molecular and kinetic properties of two acetylcholinesterases (AmAChE1 and AmAChE2) from the Western honey bee, *Apis mellifera*. Western blot analysis revealed that AmAChE2 has most of catalytic activity rather than AmAChE1, further suggesting that AmAChE2 is responsible for synaptic transmission in *A. mellifera*, in contrast to most other insects. AmAChE2 was predominately expressed in the ganglia and head containing the central nervous system (CNS), while AmAChE1 was abundantly observed not only in the CNS but also in the peripheral nervous system/non-neuronal tissues. Both AmAChEs exist as homodimers; the monomers are covalently connected via a disulfide bond under native conditions. However, AmAChE2 was associated with the cell membrane via the glycophosphatidylinositol anchor, while AmAChE1 was present as a soluble form. The two AmAChEs were functionally expressed with a baculovirus system. Kinetic analysis revealed that AmAChE2 has approximately 2,500-fold greater catalytic efficiency toward acetylthiocholine and butyrylthiocholine than AmAChE1, supporting the synaptic function of AmAChE2. In addition, AmAChE2 likely serves as the main target of the organophosphate (OP) and carbamate (CB) insecticides as judged by the lower IC_50_ values against AmAChE2 than against AmAChE1. When OP and CB insecticides were pre-incubated with a mixture of AmAChE1 and AmAChE2, a significant reduction in the inhibition of AmAChE2 was observed, suggesting a protective role of AmAChE1 against xenobiotics. Taken together, based on their tissue distribution pattern, molecular and kinetic properties, AmAChE2 plays a major role in synaptic transmission, while AmAChE1 has non-neuronal functions, including chemical defense.

## Introduction

Acetylcholinesterase (AChE, EC 3.1.1.7) is a critical enzyme in the cholinergic synapses and neuromuscular junctions of both vertebrates and invertebrates that regulates the level of the neurotransmitter acetylcholine and terminates nerve impulses [1]. AChE is a key enzyme in the insect nervous system, in which the cholinergic system is essential [2], and is the target of organophosphate (OP) and carbamate (CB) insecticides. Reduced sensitivity of AChE has been reported as one of the major resistance mechanisms against OP and CB insecticides in many arthropods [3], including the two-spotted spider mite *Tetranychus urticae* [4], the house mosquito *Culex pipiens* [5], the German cockroach *Blattella germanica* [6] and the Colorado potato beetle *Leptinotarsa decemlineata* [7].

Two cholinesterases (ChE), AChE and butyrylcholinesterase (BChE, EC 3.1.1.8), have been characterized in vertebrates, while insects possess only AChE and not BChE [8]. Studies on the evolution of ChE, including AChE and BChE, suggest that true ChEs, with highly selective substrate specificity, appear in the early bilaterians [9]. Genes for both AChE and BChE are usually present in most lineages of vertebrates, whereas duplications of the *ace* gene encoding AChE are observed in a few lineages such as nematodes, arachnids and insects. Recent studies have shown that two different *ace* loci (*ace1*, encoding AChE1, which is paralogous to *Drosophila ace*; *ace2*, encoding AChE2, which is orthologous to *Drosophila ace*) have been cloned from various insect species, such as the cotton aphid *Aphis gossypii* [10], the greenbug *Schizaphis graminum* [11], the diamondback moth *Plutella xylostella* [12] and the German cockroach *B. germanica* [13], while only one type of AChE has been discovered in cyclorrhaphan flies [14], including *Drosophila melanogaster* [15] and *Musca domestica* [16]. As indicated by phylogenetic analysis, the two *ace* genes were derived from a duplication that occurred long before the differentiation of insects, whereas the *ace1* copy was lost in Cyclorrhapha during the course of evolution [15]. Of the two insect *ace* genes, the expression of AChE1 is much greater than that of AChE2 in insects with both genes [12], [13], [17]–[19]. In addition, insects that are resistant to OP and CB insecticides possess point mutations in the *ace1* gene that are responsible for target site insensitivity [20], [21]. Based on these findings, it was proposed that AChE1 is likely the major AChE involved in synaptic transmission in insects possessing both AChE1 and AChE2 [19], [22]–[24].

In addition to the different *ace* loci expressing functionally distinct AChEs, multiple molecular forms of each AChE contribute to the functional diversification of AChEs. Several structurally distinct forms of AChE, which can be differentiated by the number and types of subunits, have been reported in both vertebrates and invertebrates [22], [25]–[29]. Insect AChE exists in three different molecular forms. The main native form is an amphiphilic dimer (G_2_m) that is attached to the plasma membrane via a glycophosphatidylinositol (GPI)-anchor [22], [28], [29]. A hydrophilic water-soluble dimer (G_2_s) can be generated from the amphiphilic dimer by proteolysis. The third form is a monomer (G_1_) that is thought to originate from the reduction of the two dimers. In addition, in *D. melanogaster*, the 55 and 18 kDa components are generated by the proteolytic cleavage of a 75 kDa precursor of AChE [30].

The Western honey bee, *Apis mellifera* L, is the most important pollinator in natural and commercial agriculture [31], [32]. In the United States, honey bees work to pollinate over 90 varieties of fruits and vegetables, including apples, avocados, blueberries, cherries, citrus crops, vine crops and almonds; these crops are valued at more than $15 billion per year. Honey bees also produce approximately $150 million in honey annually [33]. Recently, the mysterious disappearance of honey bees, called colony collapse disorder (CCD), has been reported since 2006 in the United States [34], and the global economic costs of bee decline, including lower crop yields and increased production costs, have been estimated at as high as $75 billion per year [35]. Because honey bee colonies are constantly at risk of exposure to various pesticides, including OP and CB insecticides, exposure to these insecticides may be one of the factors contributing to pollinator decline and CCD [36].

To understand the toxicity of OP and CB insecticides against honey bees, it is essential to determine whether *A. mellifera* AChE1 (AmAChE1) or AChE2 (AmAChE2) is primarily responsible for synaptic function and serves as the major target of OP and CB insecticides. In this study, the tissue distribution patterns and molecular characteristics of the two AmAChEs were investigated by native-PAGE and Western blot analysis with AChE1- and AChE2-specific antibodies. Furthermore, we expressed the two AmAChEs in Sf9 cells with a baculovirus expression system and characterized their kinetic and inhibitory properties.

## Materials and Methods

### Insects

The colonies of the Western honey bee, *A. mellifera*, that were used as sources for experimental specimens were maintained at Seoul National University. According to their behaviors and ages [37], we collected forager bees, which were older than 3 weeks of age and returned to the hive with clearly visible pollen loads on their hind legs. The collected honey bees were frozen directly with liquid nitrogen and stored at −75°C until protein extraction.

### Protein Sample Preparation

Soluble proteins were extracted from the forager heads with 0.1 M Tris-HCl (pH 7.8) buffer, while membrane-bound proteins were extracted with the same buffer supplemented with 0.5% Triton X-100. To observe the tissue distribution of AmAChE1 and AmAChE2, protein samples were prepared from six various tissues (ganglia, head, thorax, abdomen, leg, and gut) of forager bees with buffer containing Triton X-100. Proteins from various samples were extracted with an appropriate amount of buffer using a micro tissue grinder (Radnoti, Monrovia, CA, USA). The homogenates were centrifuged at 12,000×g for 15 min at 4°C. The supernatant was filtered through glasswool to remove excess lipid and stored at −75°C until use.

### Antibody Generation, PAGE and Western blotting

Sodium dodecyl sulfate (SDS)-polyacrylamide gel electrophoresis (PAGE), native-PAGE, Western blotting and AChE activity staining were conducted as previously described with some modifications [22]. Electrophoresis was performed with a vertical electrophoresis unit (Novex® mini cell, Invitrogen, Carlsbad, CA, USA). Protein preparations from various tissues (20 µg) were separated by native-PAGE gel (7.5%) in triplicate at 120 V for 90 min in a cold chamber with a continuous Tris-glycine buffer system. The gel and running buffers contained 0.5% Triton X-100 (v/v). Following native-PAGE, one set of gels was stained for AChE activity according to previously described methods [38], while the remaining two sets of gels were analyzed by Western blotting as described below.

To determine the multimer formation of AmAChEs, 20 µg of protein samples extracted from forager heads were treated with or without 14 mM ß-mercaptoethanol and separated by SDS-PAGE (4–12% gradient gel). To investigate the anchor properties of AmAChEs, proteins extracted from forager heads (20 µg) were incubated with 0.13 U of phospholipase C (PIPLC) for 20 min at 20°C. Non-treated control samples were incubated in the absence of PIPLC. After digestion, protein samples were separated on a native-PAGE gel in triplicate. After PAGE, one set of gels was stained for AChE activity, while the remaining two sets of gels were analyzed by Western blotting; one set of gels was analyzed with an AChE1-specific antibody, while the other was analyzed with an AChE2-specific antibody, as described below.

Proteins separated on the gels were transferred to Hybond-N nitrocellulose membranes (GE Healthcare, Pittsburgh, PA, USA) by electroblotting. After blocking in PBS buffer containing 0.1% Tween-20 (PBST) and 5% fat-free dry milk for 1 h at room temperature, the nitrocellulose membrane sheets were incubated for 3 h at room temperature or overnight at 4°C with primary antibodies (anti-AChE1 or anti-AChE2) [22]. Anti-AChE1 and anti-AChE2 polyclonal antibodies were generated as described previously [22]. The membranes were then incubated with horseradish-peroxidase conjugated anti-rabbit IgG secondary antibody (Pierce Bio-Technology, Rockford, IL, USA) for 1 h. The antigen-antibody complex on the bands was visualized with a chemiluminescence kit according to the manufacturer's instructions (Santa Cruz Biotechnology, Santa Cruz, CA, USA).

### *In vitro* Expression of AmAChE1 and AmAChE2 with a Baculovirus Expression System

Total RNA was extracted from forager heads with TRI reagent (MRC, Cincinnati, OH, USA) as described by the manufacturer. Following extraction, the total RNA was treated with DNaseI (TAKARA Korea Biomedical Inc., Seoul, Korea) at 37°C for 30 min and concentrated with 3 M sodium acetate. First strand cDNA was synthesized from the DNaseI-treated total RNA with Superscript III reverse transcriptase (Invitrogen) at 55°C for 1 h by priming with oligo dT, and the RNA strand was then removed by incubation with RNase H (Invitrogen) at 37°C for 20 min. The complete cDNA fragments encoding *A. mellifera ace1* and *ace2* (*AmAce*1 and *AmAce2*, respective GenBank accession numbers XM393751 and AF213012) were amplified by Advantage Taq (Clontech, Palo Alto, CA, USA) with gene-specific primers (Table S1) and directly cloned into the pGEM®-T easy vector (Promega, Madison, MU, USA). Partial fragments of *AmAce*1 and *AmAce*2 with truncated C-terminal hydrophobic regions were amplified by ExTaq (Takara, Japan) at 95°C for 2 min, (95°C for 30 s, 65°C for 30 s, 72°C for 2 min) × 5 cycles, (95°C for 30 s, 60°C for 30 s, 72°C for 2 min) × 30 cycles and 72°C for 2 min with gene-specific primers containing restriction enzyme sites (Table S1) from respective full ORF clones. The amplified *AmAce1 and AmAce*2 DNA fragments were digested with *Xba*I and *Sac*I (Koschem, Korea) and inserted into pBacPAK8 (Clontech) that had been digested with the same restriction endonucleases. Recombinant baculoviruses expressing AmAChE1 and AmAChE2 in SF9 cells were generated as described previously [22]. Virus-infected cells were incubated for 84 h at 27°C. Protein samples were collected by centrifugation and concentrated with an Ultra Amicon YM-30 (Millipore, Bedford, MA, USA). Protein concentrations were determined by the Bradford method with bovine serum albumin as the standard protein [39]; the proteins were then stored at −75°C until use.

### Kinetics and Inhibition of AmAChEs

The enzyme assay for AmAChEs expressed in Sf9 cells was performed with 8 different concentrations (0.05 to 1 mM) of acetylthiocholine iodide (ATChI) and butyrylthiocholine iodine (BTChI) according to previously described methods with some modifications [40], [41]. To measure enzyme kinetics, 15 µl of culture supernatant containing 5 µg of one of the two AmAChEs as enzyme sources was added to each well containing 85 µl of substrate mixture in the presence of 0.4 mM 5,5'-dithiobis-(2-nitrobenzoic acid) (DTNB); the wild type virus was used as a blank control. The reaction was monitored at 412 nm for 5 min with 10-sec intervals with a Soft Max® Pro5 microplate reader (Molecular Devices, Menlo Park, CA) at 30°C. Michaelis-Menten constants (K_m_) and maximal velocity (V_max_) values for each substrate were determined by Lineweaver-Burk plot.

AmAChE inhibition was assayed at 7 different concentrations (10^−9^ to 10^−3^ M) of each of three inhibitors (BW284C51, eserine and Iso-OMPA), three organophosphates (DDVP, malaoxon and paraoxon) and four carbamates (aldicarb, carbaryl, carbofuran and propoxur), according to previously described methods with some modifications [42]. Due to high sensitivity of AmAChEs to chlorpyrifos-oxon, 10^−12^ to 10^−6^ M of chlorpyrifos-oxon was used for inhibition assay. Five µg of each AmAChE as enzyme source was added to each well containing the substrate mixture of 1 mM ATChI and 0.4 mM DTNB and various concentrations of inhibitors to initiate enzyme reaction. Upon initiation of reaction, AmAChE activities were recorded at 412 nm for 3 min with 10-sec intervals using a microplate reader as above. To investigate the scavenger effects of AmAChE1 on the inhibition of AmAChE2, an AmAChE mixture was inhibited with 7 different concentrations of each of two OP (chlorpyrifos -oxon and malaoxon) and CB (carbofuran and propoxur) according to previously described methods with some modification [43]; the molar ratio of AmAChE1:AmAChE2 was 2∶1 (4∶2 µg) based on the their expressed protein levels in forager bees. The inhibitors were pre-incubated with AmAChE1 for 30 min at 30°C, and AmAChE2 was then added. The same molar amount of BSA instead of AmAChE1 was used as a control. AmAChE activities in the presence of 1 mM ATChI, 0.4 mM DTNB and various concentrations of inhibitors were recorded as above. Five consecutive concentrations of each inhibitor exhibiting a relatively linear range of inhibition were selected for the calculation of median inhibition concentration (IC_50_). The IC_50_ for each inhibitor was determined based on log-concentration versus probit (% inhibition) regression analysis using SPSS for Windows version 20.0 K (SPSS Inc., Chicago, IL).

### 3D Structure Modeling, Hydrophobicity and GPI-anchor Prediction

The 3D structure analyses of AmAChE1 and AmAChE2 were performed with the Automated Comparative Protein Modeling Server of SWISS-MODEL (http://swissmodel.expasy.org/) using human BChE and *D. melanogaster* AChE as the respective templates. Structure comparisons between AmAChEs were performed with UCSF Chimera MatchMaker ver. 1.4 (University of California, CA). The models were visualized and modified with Swiss PDB viewer 4.0.1 (Swiss Institute of Bioinformatics, Lausanne, Switzerland). The hydrophobicities of AmAChE1 and AmAChE2 were predicted with ProtScale of the ExPASy Proteomics Server (http://expasy.org/cgi-bin/protscale.pl). The potential GPI-anchor sequences of two AmAChEs were predicted with GPI-SOM of the ExPASy Proteomics Server (http://gpi.unibe.ch/).

## Results

### Expression Patterns of AmAChEs in Various Tissues

**Figure 1:**
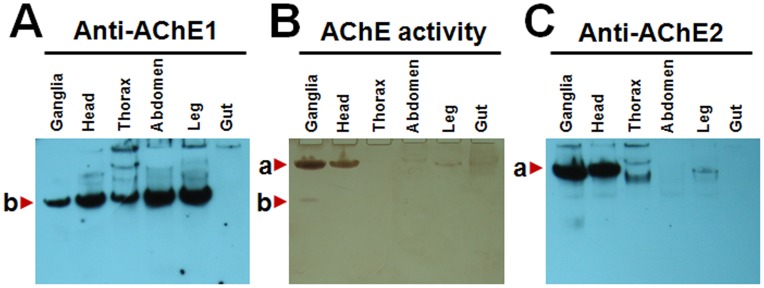
Tissue distribution of AmAChE1 and AmAChE2 as assessed by native polyacrylamide gel electrophoresis and Western blot analysis. Protein samples (20 µg) from various tissues were loaded onto a 7.5% polyacrylamide gel and run for 90 min at 120 V. After electrophoresis, one gel was stained for activity with acetylthiocholine iodide as a substrate (B). The other gels were analyzed by Western blot with anti-AChE1 (A) or anti-AChE2 (C) polyclonal antibodies.

To determine the tissue-specific expression profiles of two AmAChEs, native-PAGE was performed on proteins extracted from six tissues (thoracic ganglia, head, thorax, abdomen, legs and gut) of forager bees, and their AChE activities were visualized by activity staining (Fig. 1B). The AmAChEs were concentrated in the ganglia and heads containing the central nervous system (CNS) (band ‘a’), but little activity was detected in the peripheral nervous system (PNS) such as in the thorax, abdomen, legs and gut.

Western blot analysis revealed that the main AChE activity is actually associated with AmAChE2 (Fig. 1C, band ‘a’), and AmAChE2 was more predominantly expressed in the CNS than in other tissues. By contrast, although AmAChE1 activity was faintly detected in ganglia (Fig. 1B, band ‘b’), AmAChE1 was not detectable in other tissues. Nevertheless, AmAChE1 seemed to be abundantly expressed not only in the CNS but also in the PNS such as in the thorax, abdomen and legs, as judged by Western blot analysis (Fig. 1A, band ‘b’).

### Molecular Characterization of AmAChEs

**Figure 2:**
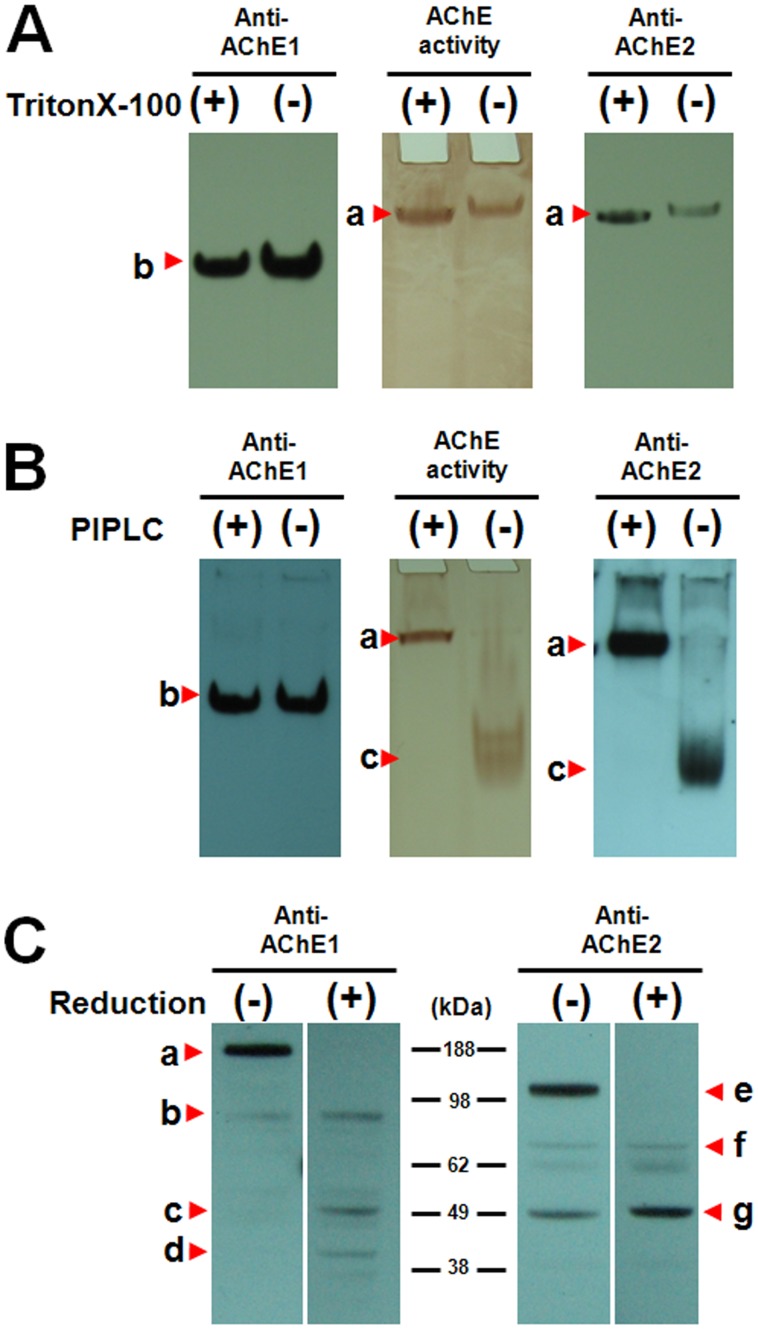
Molecular characterization of AmAChE1 and AmAChE2 by polyacrylamide gel electrophoresis and Western blot analysis. Protein samples were extracted from the heads of forager honey bees with 0.1 M Tris-HCl buffer in the presence or absence of 0.5% Triton X-100 to determine the soluble nature of the AmAChEs (A). To investigate the GPI-anchor properties of the AmAChEs, protein samples were treated with PIPLC (B). Protein samples were mixed with or without β-mercaptoethanol and separated by sodium dodecyl sulfate polyacrylamide gel electrophoresis to determine the multimer formation of AmAChE1 and AmAChE2 (C).

Crude protein was extracted from forager heads with 0.1 M Tris-HCl buffer in the presence or absence of Triton X-100 to determine the molecular formations and soluble nature of AmAChE1 and AmAChE2. When the protein was extracted with Triton X-100-containing buffer, both AmAChE1 and AmAChE2 were strongly detected (Fig. 2A, see the Triton X-100 (+) lanes). In the absence of Triton X-100, the AmAChE1 band was still clearly observed, while AmAChE2 was faintly detected (Fig. 2A, see the Triton X-100 (−) lanes), suggesting the membrane-anchored nature of AmAChE2. To confirm the membrane-anchored properties of AmAChEs, AmAChEs extracted from forager heads were digested with PIPLC. After PIPLC treatment, no molecular changes were observed in AmAChE1, whereas amphiphilic AmAChE2 was completely converted to the hydrophilic form (Fig. 2B). This result indicates that AmAChE2 is associated with the membrane via a GPI-anchor, while AmAChE1 is present in a soluble form due to the absence of a GPI-anchor, as indicated by PIPLC treatment as well as protein extraction in the absence of Triton X-100.

The molecular masses of AmAChEs were analyzed by SDS-PAGE in the presence or absence of β-mercaptoethanol reduction (Fig. 2C). Under non-reducing conditions, bands of approximately 185 kDa (band ‘a’) and 140 kDa (band ‘e’) were strongly visualized as putative dimers, while bands of 88 kDa (band ‘b’) and 79 kDa (band ‘f’) were faintly detected as putative monomers by the respective AmAChE1- and AChE2-specific antibodies (see reduction (-) lanes). In addition to the putative dimeric form, a band of approximately 50 kDa representing cleaved monomer (band ‘g’) was also clearly detected for AmAChE2. This finding suggests that dimers are the predominant forms of both AmAChEs under native conditions and that the cleaved monomer is also abundantly present in AmAChE2. After reduction with β-mercaptoethanol, the relative quantities of the other forms, such as the monomers (88 kDa band ‘b’ for AmAChE1 versus 70 kDa band ‘f’ for AmAChE2) and cleaved forms (48 kDa band ‘c’ and 40 kDa band ‘d’ for AmAChE1 vs. 50 kDa band ‘g’ for AmAChE2), increased, while the dimeric forms of both AmAChEs disappeared, supporting the presence of a disulfide bond in the dimer conformation. As indicated by the amino acid sequence alignment with *Drosophila* and German cockroach AChEs (Fig. S1B), the presence of cysteine at the C-terminal region of several insect AChEs also supports the role of a disulfide bond connection in dimer formation for both AmAChE1 and AmAChE2.

### Kinetic Properties of AmAChEs

As judged by Western blotting with AChE1- and AChE2-specific antibodies, two types of AmAChEs were successfully expressed with the recombinant baculovirus expression system. *In vitro* expressed AmAChE2 demonstrated strong AChE activity, whereas AmAChE1 activity was barely detectable, as observed in the protein samples from the heads of forager honey bees (Fig. S2A). In addition, the estimated molecular masses of both AmAChEs (approximately 70 kDa) were confirmed by Western blotting following SDS-PAGE (Fig. S2B).

Two cholinesterase substrates, ATChI and BTChI, were used to study the kinetic properties of the two AmAChEs. The K_m_ and V_max_ values were calculated by double-reciprocal plots (Fig. S3). The kinetic parameters for the two substrates are presented in Table 1. AmAChE1 and AmAChE2 exhibited 3.8- and 4-fold higher catalytic efficiency [Maximal velocity/Michaelis-Menten constants (V_max_/K_m_)], respectively, toward ATChI than BTChI, confirming the typical substrate specificities of AChEs. In a cross-enzyme comparison of AmAChE1 and AmAChE2, AmAChE2 showed lower K_m_ values than AmAChE1 (1.28- and 12.5-fold for ATChI and BTChI, respectively), demonstrating higher substrate affinity compared to AmAChE1. AmAChE2 also exhibited approximately 200-fold higher V_max_ values for both ATChI and BTChI than did AmAChE1. Taken together, AmAChE2 exhibited approximately 2,500- and 2,400-fold higher catalytic efficiencies toward ATChI and BTChI, respectively, than did AmAChE1, indicating that AmAChE2 is a much more efficient enzyme than AmAChE1. The ratio V_max_(BTChI)/V_max_(ATChI) was 0.51 and 0.5 for AmAChE1 and AmAChE2, respectively, indicating that the substrate spectrums of AmAChE1 and AmAChE2 were similar.

**Table 1:** Kinetic properties of recombinant two AmAChEs in hydrolyzing various substrates*.

Substrate	Kinetic property	AmAChE1	AmChE2	Ratio†
ATChI	V_max_(mM/min/mg protein)	0.308±0.081	60.8±0.686	197
	K_m_ (mM)	1.14±0.381	0.089±0.011	0.078
	V_max_/K_m_ (Ratio)	0.269	684	2543
				
BTChI	V_max_ (mM/min/mg protein)	0.157±0.029	30.4±3.91	194
	K_m_ (mM)	2.23±0.279	0.179±0.029	0.08
	V_max_/K_m_ (Ratio)	0.07	170	2429
				
Substrate specificity	V_max_ (BTChI)/V_max_ (ATChI)	0.51	0.5	

* Results are reported as the mean ± SD (n  = 3).

† AmAce2/AmAce1.

### Inhibitory Properties of AmAChEs

The inhibitory properties of AmAChEs were determined with various concentrations of three reversible cholinesterase-specific inhibitors, four OPs and four CBs (Fig. S4 and Table 2). Both AmAChEs were effectively inhibited by BW284C51 and eserine but not by Iso-OMPA, a BChE-specific inhibitor, suggesting that both enzymes retain typical features of AChEs [44]. BW284C51 similarly inhibited both AmAChEs, while AmAChE2 was 16-fold more sensitive to eserine than was AmAChE2, as judged by IC_50_ values. In the inhibition assay with OPs and CBs, AmAChE2 was, in general, much more sensitive to these insecticides than was AmAChE1 (Table 2). As indicated by the IC_50_ values of the OPs, AmAChE2 was approximately 4-, 7-, 3- and 45-fold more sensitive to chlorpyrifos, DDVP, malaoxon and paraoxon, respectively. The CBs also inhibited AmAChE2 more effectively than did AmAChE1 (3,500-, 19- and 3-fold for carbaryl, carbofuran and propoxur, respectively), whereas neither AmAChE was inhibited by aldicarb. In a cross-inhibitor comparison of OPs and CBs, OPs generally exhibited much lower IC_50_ values than CBs, suggesting that OPs are more efficient inhibitors of both AmAChE1 and AmAChE2 than CBs.

**Table 2:** IC_50_ (M) values of different inhibitors of two recombinant AmAChEs*.

Inhibitor	AmAce1	AmAce2	Ratio of IC_50_ †
BW284C51	(4.33±0.58) × 10^−9^	(2.67±0.21) × 10^−9^	1.68
Eserin	(1.73±0.41) × 10^−5^	(1.09±0.03) × 10^−6^	15.89
Iso_OMPA	n/a	n/a	n/a
Chlorpyrifos oxon	(6.12±0.25) × 10^−7^	(1.53±0.19) × 10^−7^	3.99
DDVP	(3.53±0.15) × 10^−4^	(5.03±0.40) × 10^−5^	7.01
Malaoxon	(9.19±0.43) × 10^−6^	(3.26±0.29) × 10^−6^	2.82
Paraoxon	(4.58±0.11) × 10^−5^	(1.01±0.04) × 10^−6^	45.4
Aldicarb	n/a	n/a	n/a
Carbaryl	(1.20±0.51) × 10^−3^	(3.43±0.69) × 10^−7^	3.49 × 10^3^
Carbofuran	(1.69±0.24) × 10^−5^	(9.02±0.19) × 10^−7^	18.78
Propoxur	(1.85±0.12) × 10^−4^	(5.33±0.49) × 10^−5^	3.48

* Results are reported as the mean ± SD (n  = 3).

† AmAce1/AmAce2.

### Reduction of AmAChE2 Inhibition by the Presence of AmAChE1

**Figure 3:**
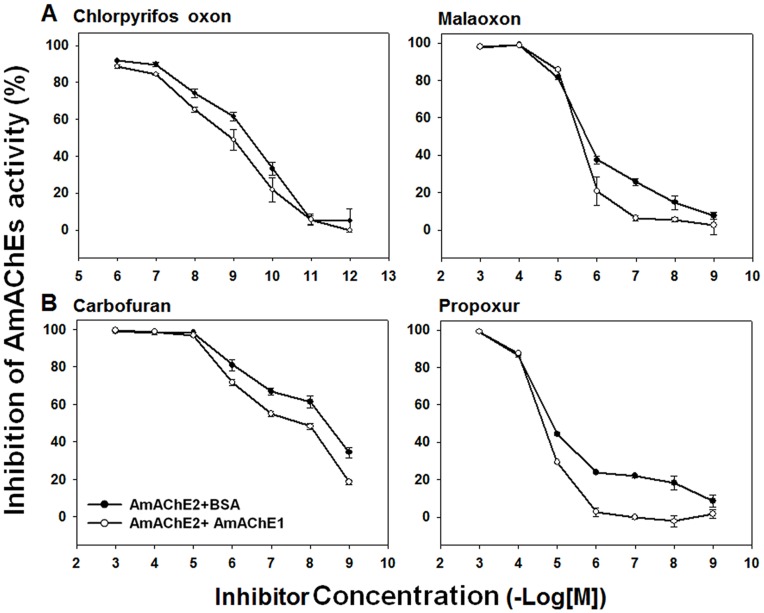
Inhibition of AmAChE2 by two organophosphates (A) and two carbamates (B) in the presence (○) or absence of AmAChE1 ( •**).** Inhibitors were pre-incubated with AmAChE1 for 30 min at 30°C, and AmAChE2 was then added. The same molar amount of BSA in place of AmAChE1 was used as a control.

To examine the physiological function of AmAChE1, the inhibition rates of two OPs and two CBs against AmAChE2, the major catalytic enzyme in the honey bee, were measured in the presence or absence of AmAChE1 (Fig. 3). As judged by the IC_50_ values (Table S2), the overall inhibition was reduced 2.3- to 4.5-fold when inhibitors were pre-incubated with AmAChE1 and then added to AmAChE2 compared to pre-incubation with BSA, suggesting that one of the physiological functions of AmAChE1 may be chemical defense against xenobiotics, perhaps by sequestering inhibitors.

### Three-dimensional Structure Modeling

**Figure 4:**
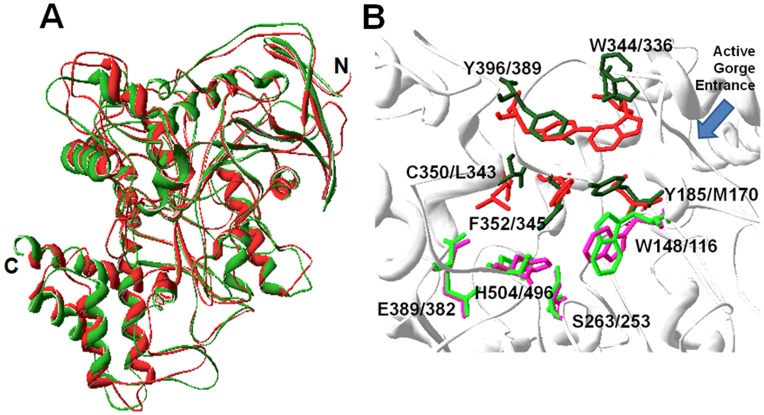
Comparison of the predicted three-dimensional (3D) structures of AmAChE1 (green color) and AmAChE2 (red color). The superimposed 3D structures (A) and zoom-in views (B) of the two AmAChEs were compared. The positions of amino acid residues involved in the formation of the catalytic triads, choline-binding sites and peripheral anionic sites are indicated (AmAChE1/AmAChE2) (B).

The 3D structures of AmAChE1 and AmAChE2 were predicted with Swiss Model using the structures of human BChE and *D. melanogaster* AChE as the respective templates (Fig. 4). In the structure comparison between AmAChE1 and AmAChE2, most of the α-helix and β-stranded sheets were highly overlapped, demonstrating that the overall structures of the two AmAChEs were similarly folded (Fig. 4A). When the catalytic gorge structures of the two AmAChEs were merged, the choline-binding site (W148) and the catalytic triad (S263, E389 and H504) of AmAChE1 closely overlapped those of AmAChE2 (Fig. 4B). However, the shape of the peripheral anionic site (PAS) changed because of differences in two amino acid residues: Y185 in AmAChE1 versus M170 in AmAChE2 and C350 in AmAChE1 versus L343 in AmAChE2. In addition, the angles of the choline-binding site (W344/336) at the entrance to the active gorge in AmAChE1 and AmAChE2 differed by approximately 90°, suggesting that the differences in the PAS conformation may be responsible for the differences in the substrate and inhibition kinetics of AmAChE1 and AmAChE2.

## Discussion

**Figure 5:**
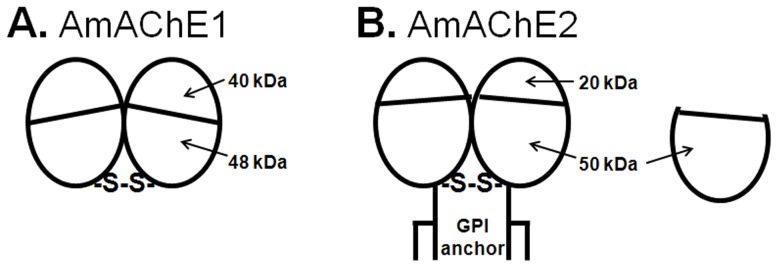
Schematic diagrams of the molecular structures of AmAChE1 (A) and AmAChE2 (B). One type of AmAChE1 is predominately expressed (A), while two different molecular polymorphisms of AmAChE2 were abundantly observed (B).

In this study, we determined the molecular polymorphisms of AmAChEs and the identity of the major catalytic enzyme by native-PAGE and Western blot analyses with AChE1- and AChE2-specific antibodies. The most predominant molecular form of both AmAChEs was a dimer formed by an intersubunit disulfide bridge (Fig. 2C), as observed in *Drosophila* [30], German cockroach [22] and *Torpedo* AChEs [45]. In addition, amphiphilic dimers of AmAChE2 were completely converted to hydrophilic dimers by PIPLC treatment, whereas the molecular migration pattern of AmAChE1 was unchanged (Fig. 2B). According to the hydrophobicity plot (Fig. S1A) and GPI-anchor prediction (Fig. S1B), AmAChE2 is predicted to have GPI-anchor sequences at the hydrophobic C-terminal region. By contrast, no GPI-anchor sequence was predicted in the AmAChE1 sequence, and the C-terminal region of AmAChE1 is predicted to be hydrophilic, supporting the soluble and membrane-anchored properties of AmAChE1 and AmAChE2, respectively. Based on the molecular characteristics of AmAChE1 and AmAChE2, schematic models of the structures of these enzymes were generated (Fig. 5). The monomer of both AmAChE1 and AmAChE2 is composed of two small subunits (40 and 48 kDa for AmAChE1 vs. 20 and 50 kDa for AmAChE2), and two monomers are linked by a disulfide bond at the C-terminal regions. AmAChE1 predominantly exists as a soluble dimer, while the most abundant molecular form of AmAChE2 appeared to be attached to the membrane via a GPI-anchor. In addition, a small fraction of AmAChE2 is present as a 50-kDa subunit under native conditions (Fig. 5B).

Contrary to the general idea that AChE1 is the major synaptic enzyme in most insects having both AChE1 and AChE2 studied to date [19], [22], AmAChE2 appears to function as the main synaptic enzyme in the honey bee, as judged by the migration patterns of the AChE activity bands and the protein bands detected by Western blotting. In addition, the distribution of AmAChE2 was greater in tissues associated with the CNS, such as the ganglia and head, while AmAChE1 was strongly detected not only in the CNS but also in the PNS/non-neuronal tissues, such as the thorax, abdomen and legs (Fig 1). In *Drosophila* [1], [29], [46], the membrane-bound form of AChE exhibits greater AChE activity than does the soluble AChE. Moreover, the AChE activities of the fruit fly and house fly were reduced after treatment with PIPLC or β-mercaptoethanol to convert membrane-bound AChE to the soluble form [16], [28]. Likewise, as judged by native-PAGE in conjunction with Western blotting, the enzymatic activity of membrane-bound AmAChE2 is much greater than that of the soluble AmAChE1, which has negligible enzymatic activity. In fact, a comparison of the catalytic properties of *in vitro*-expressed AmAChE1 and AmAChE2 also strongly confirmed that AChE activity in the honey bee primarily originates from AmAChE2, which possesses approximately 2,500-fold higher catalytic efficiency than AmAChE1 (Table 1). Furthermore, in the inhibition assays, AmAChE2 was much more sensitive to inhibitors in general, as indicated by the IC_50_ values (Table 2), implying that the major target molecule of OP and CB insecticides is AmAChE2, which functions in neurotransmission. Compared to other insecticides, chlropyrifos-oxon exhibited much lower IC_50_ values to AmAChE2 (Table 2). Interestingly, it was reported that LD_50_ value of chlorpyrifos to the honey bee was generally much lower than other insecticides [32]. Taken together, the high sensitivity of AmAmChE2 to chlorpyrifos-oxon may be mainly responsible for the high toxicity of chlorpyrifos to the honey bee. In a previous study, the AmAChE responsible for the majority of AChE activity was cloned, and its expression pattern was evaluated as a function of the worker bee’s development state, although the major AChE in the honey bee was not assigned to one of the two different types of AChEs [47]. The AmAChE cDNA sequence (GenBank accession number AF213012) used in that study is the same as that of AChE2, also supporting that *A. mellifera* expresses AChE2 as the major catalytic enzyme.

As shown by 3D structure comparison, the catalytic triad conformations of the two AmAChEs were similar, with the exception of some predicted structural differences at the active gorge entrance (Fig. 4). Most notably, the aromatic side chain angles of the choline-binding sites (W344 in AmAChE1 vs. W336 in AmAChE2) differed by approximately 90°. In addition, Y185 and C350 in AmAChE1 were replaced by M170 and L343 in AmAChE2, respectively, altering the topology of the gorge entrance. These amino acid replacements were highly similar to those of *B. germanica*, and the catalytic efficiency of cockroach AChE2 was also higher than that of AChE1 [22]. As observed in *B. germanica*, these conformational differences between the two AmAChEs appear to be responsible for the kinetic and inhibitory properties.

Considering that a large fraction of AmAChE1 exists as a soluble form in the PNS (Fig. 1) and the enzymatic activity of AmAChE1 is lower than that of AmAChE2 (Table 1), AmAChE1 seems to play other non-neuronal functions, in contrast to AmAChE2. As in the case of vertebrate BChE [48] and nematode AChE [43], we investigated the hypothesis that AmAChE1 plays a role in chemical defense against xenobiotics. When OPs and CBs were pre-incubated with AmAChE1 for 30 min prior to the addition of AmAChE2, the inhibition of AmAChE2 by the insecticides was reduced significantly (Fig. 3), implying that AmAChE1 functions as a ubiquitous sequestration protein rather than as a specific hydrolase by binding nonspecifically to a variety of xenobiotics, particularly OP and CB insecticides. This physiological property of AmAmChE1 highly resembles that of pinewood nematode AChE3 (BxAChE3) [43]. Among three BxAChEs, BxAChE3 showed the highest expression level and lowest catalytic efficiency toward substrates [49]. In addition, a significant reduction in the inhibition of AChE by insecticides was observed in the presence of BxAChE3, indicating that BxAChE3 protects BxAChE1 and BxAChE2 [43] from potential inhibitors. The bio-scavenging function of ChE has been broadly investigated in vertebrate BChEs. In pigs and monkeys, administration of BChE increases LD_50_ values against nerve agents, demonstrating that BChE can function as a biological scavenger to provide significant protection against the behavioral and lethal effects of nerve agent intoxication before these agents arrive at their target sites [50]–[52]. This soluble enzyme is also abundantly observed in non-neuronal tissues, such as plasma, liver, lung and intestine [53]. These results support the physiological function of soluble AmAChE1 as a bio-scavenger that is predominantly expressed in both neuronal (most likely the extracellular space in the blood-brain barrier) and non-neuronal tissues (most likely the hemocoel) without high catalytic efficiency. In fact, although the honey bee is considered to be particularly sensitive to insecticides, it was no more sensitive than other insect species to various insecticides including CBs, OPs, nicotinoids, organochlorines, pyrethroids and others [32], which may be due to the bio-scavenging function of AmAChE1. In addition to such putative bio-scavenging function, a recent study suggested that a non-neuronal AChE is likely involved in various physiological functions, such as female reproduction, embryo development and growth of offspring in *Tribolium castaneum* [23], [24].

In summary, AmAChE2 was present at high concentrations in the CNS, such as the head and ganglia, and has high catalytic efficiency. Based on its tissue distribution, membrane-anchoring properties and high catalytic efficiency, AmAChE2 appears to play a major role in postsynaptic transmission. Inhibition studies with OPs and CBs demonstrated that AmAChE2 is more sensitive to inhibitors than AmAChE1, supporting the role of AmAChE2 as the major target molecule of OP and CB insecticides. Although AmAChE1 has low enzyme activity, soluble dimeric AmAChE1 was also abundant in non-neuronal tissues. In particular, the ability of AmAChE1 to reduce the inhibition of AmAChE2 by insecticides strongly suggests a physiological function of AmAChE1 as a bio-scavenger that provides a chemical defense against xenobiotics.

## Supporting Information

Figure S1 **Hydrophobicity prediction (A) and GPI-anchor prediction (B) of AmAChE1 and AmAChE2.** Red ovals indicate the C-terminal region of each of the AmAChEs (A). The predicted GPI-anchor sites and the cysteine residues that form the disulfide bond in each of the AChEs are represented by red arrows and red circles, respectively (B).
(TIF)

Figure S2 **Expression of recombinant AmAChE1 and AmAChE with a baculovirus expression system.** The expression of AmAChE1 and AmAChE2 was confirmed by AChE activity staining and Western blotting following native polyacrylamide gel electrophoresis (A) and sodium dodecyl sulfate polyacrylamide gel electrophoresis (B).
(TIF)

Figure S3 **Double-reciprocal plots for AmAChE1 (A) and AmAChE2 (B) for the calculation of K_m_ and V_max_.** The straight lines were generated by plotting 1/v versus 1/[S]; the slope indicates K_m_/V_max_, and the intercept of the x-axis (1/[S]) indicates −1/K_m_.
(TIF)

Figure S4 **Inhibition of AmAChE1 (**•**) and AmAChE2 (○) by cholinesterase-specific inhibitors (A), organophosphates (B) and carbamates (C).** The results are the mean of three determinations (n = 3). Vertical bars indicate standard deviations.
(TIF)

Table S1 **Primers used for the in vitro expression of AmAChE1 and AmAChE2 with a baculovirus expression system.**
(DOCX)

Table S2 **IC_50_ (M) values of different inhibitors of recombinant AmAChE2 with or without pre-incubation with AmAChE1.**
(DOCX)
