# Supplementary figures and images for: Molecular and Kinetic Properties of Two Acetylcholinesterases from the Western Honey Bee, Apis mellifera
Source: PLoS One. 2012 Nov 7;7(11):e48838. doi: 10.1371/journal.pone.0048838 (PMC3492254; doi:10.1371/journal.pone.0048838)

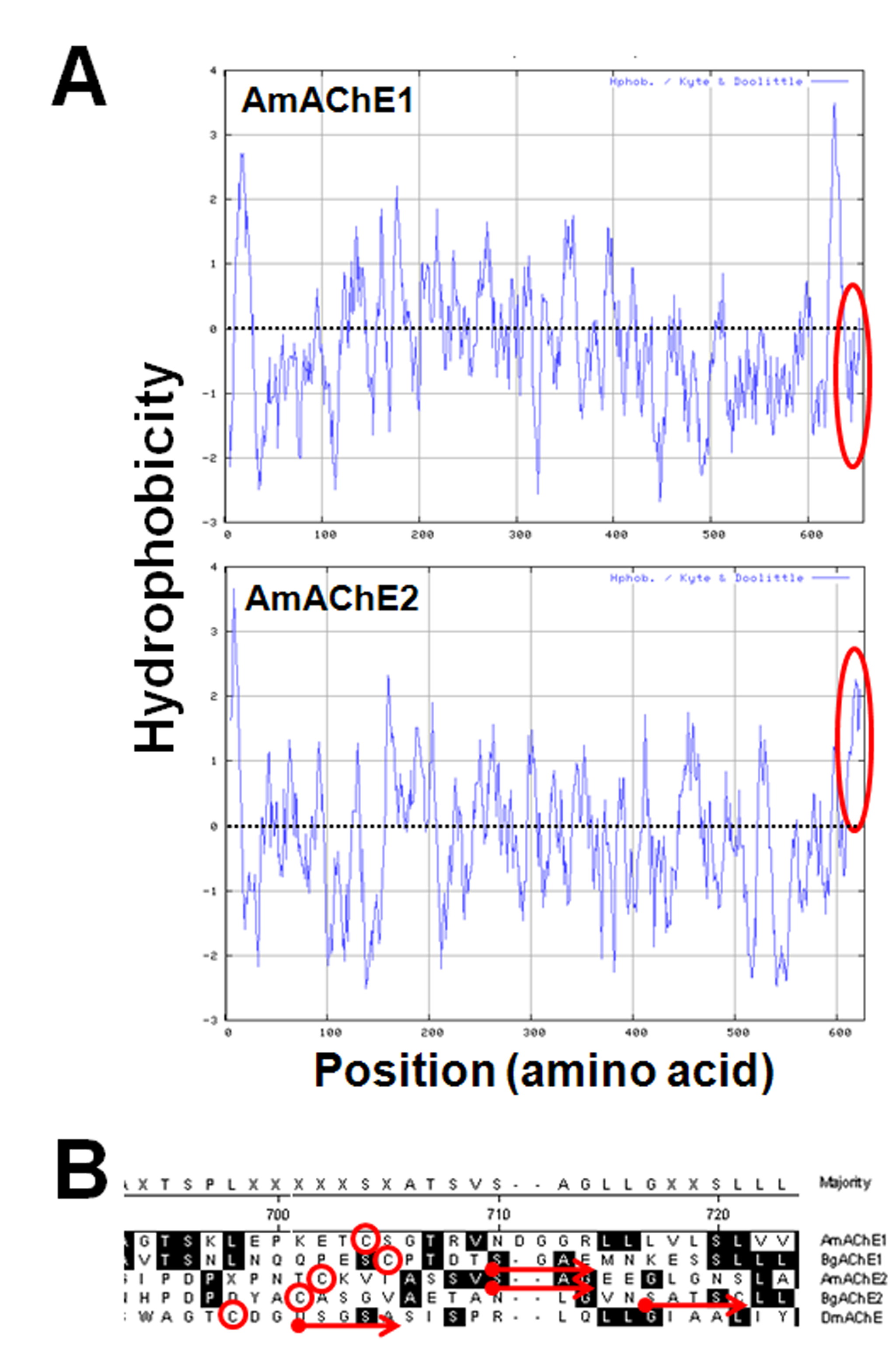

Supplement: Figure S1 — Hydrophobicity prediction (A) and GPI-anchor prediction (B) of AmAChE1 and AmAChE2. Red ovals indicate the C-terminal region of each of the AmAChEs (A). The predicted GPI-anchor sites and the cysteine residues that form the disulfide bond in each of the AChEs are represented by red arrows and red circles, respectively (B). (TIF) [file pone.0048838.s001.tif]

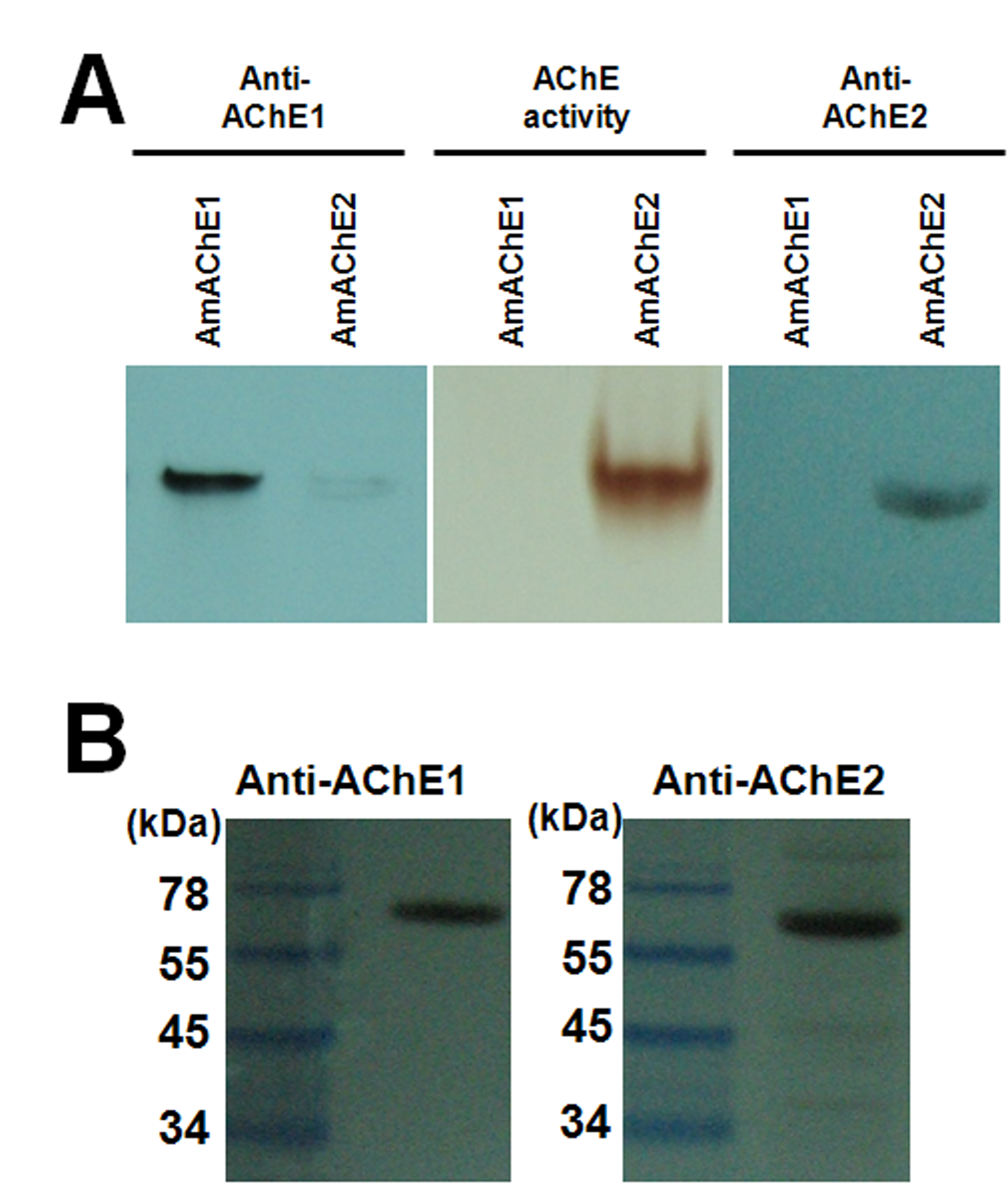

Supplement: Figure S2 — Expression of recombinant AmAChE1 and AmAChE with a baculovirus expression system. The expression of AmAChE1 and AmAChE2 was confirmed by AChE activity staining and Western blotting following native polyacrylamide gel electrophoresis (A) and sodium dodecyl sulfate polyacrylamide gel electrophoresis (B). (TIF) [file pone.0048838.s002.tif]

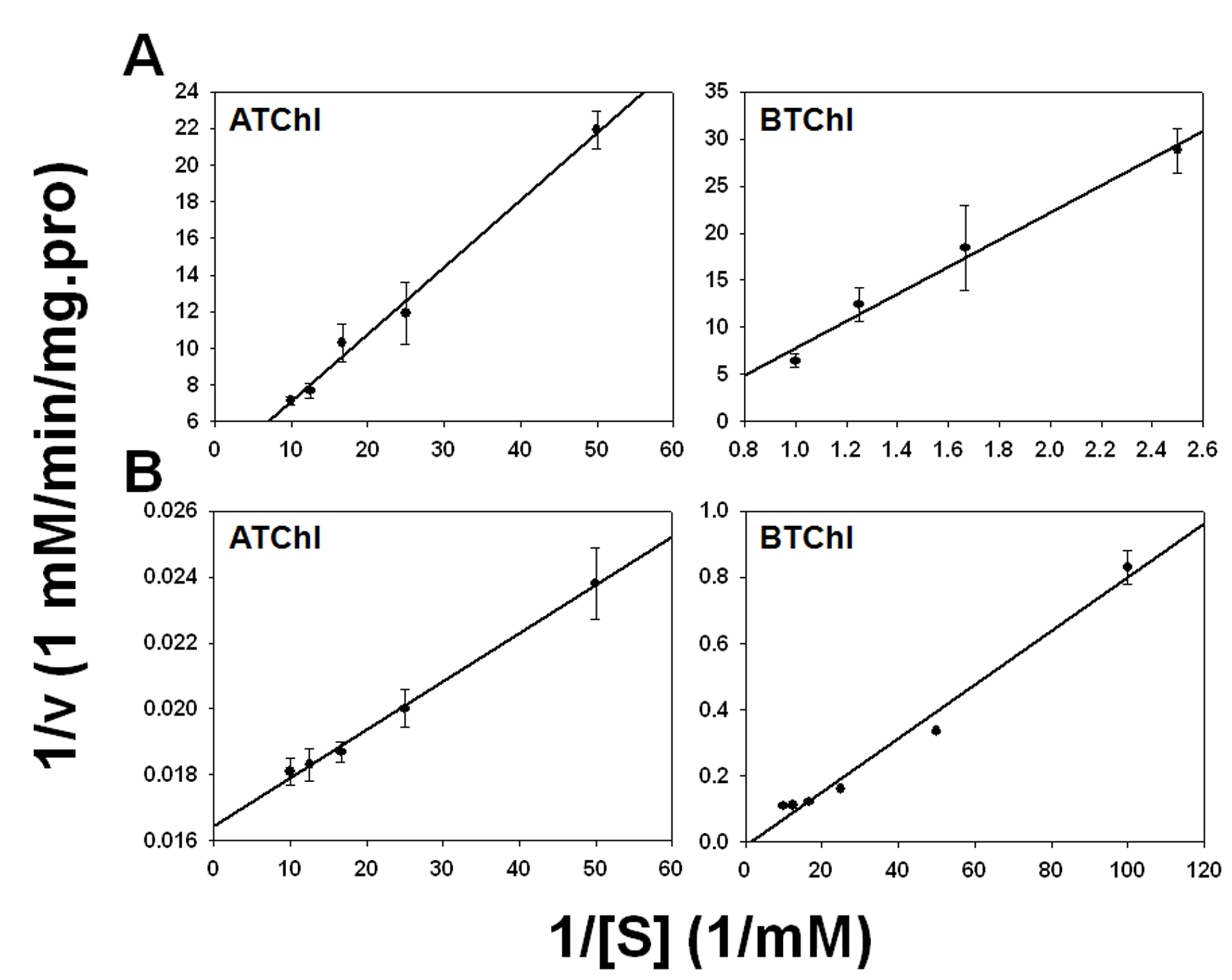

Supplement: Figure S3 — Double-reciprocal plots for AmAChE1 (A) and AmAChE2 (B) for the calculation of Km and Vmax. The straight lines were generated by plotting 1/v versus 1/[S]; the slope indicates Km/Vmax, and the intercept of the x-axis (1/[S]) indicates −1/Km. (TIF) [file pone.0048838.s003.tif]

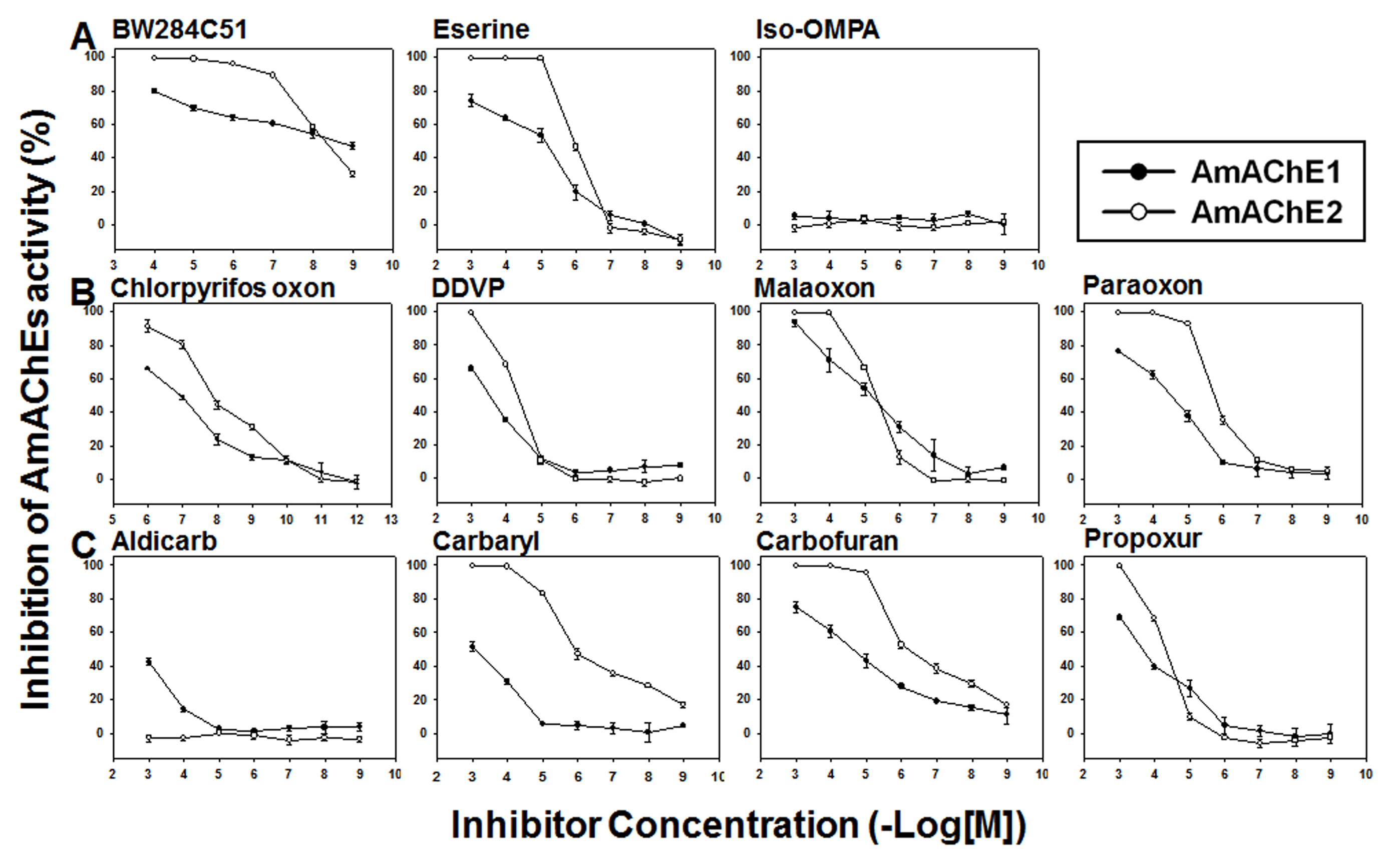

Supplement: Figure S4 — Inhibition of AmAChE1 (•) and AmAChE2 (○) by cholinesterase-specific inhibitors (A), organophosphates (B) and carbamates (C). The results are the mean of three determinations (n = 3). Vertical bars indicate standard deviations. (TIF) [file pone.0048838.s004.tif]
