# Supplementary material for: Molecular and Kinetic Properties of Two Acetylcholinesterases from the Western Honey Bee, Apis mellifera
Source: PLoS One. 2012 Nov 7;7(11):e48838. doi: 10.1371/journal.pone.0048838 (PMC3492254; doi:10.1371/journal.pone.0048838)
Supplement: Table S1 — Primers used for the in vitro expression of AmAChE1 and AmAChE2 with a baculovirus expression system. (DOCX) [file pone.0048838.s005.docx]

**Table S1. Primers used for in vitro expression of AmAChE1 and AmAChE2 using baculovirus expression system^*^.**

| Name | sequence | Purpose |
| --- | --- | --- |
| 5’AmAChE1_end | ATGCCGAGAAACCGAAATTCCTTGACC | AmAChE1 and AmAChE2 ORF cloning |
| 3’AmAChE1_end | CTAGACGAACCCTTTCGGATCGAATATC |  |
| 5’AmAChE2_end | ATGACTACGAGGATCCTACTCCTGTTC |  |
| 3’AmAChE2_end | TTAAATCACTCTCGACAGGACAAGGATC |  |
| 5’AmAChE1_XbaI | TCTAGAATGCCGAGAAACCGAAATTCCTTGACCAAG | Partial fragment cloning into pBacPAK8 |
| 3’AmAChE1_SacI | GAGCTCAGTGCCGGCGACCAGTTGGGGAAGATA |  |
| 5’AmAChE2_XbaI | TCTAGAATGACTACGAGGATCCTACTCCTGTTCTTC |  |
| 3’AmAChE2_SacI | GAGCTCTGGTATTCCTTTCAACTTTGGTAGAAATTCG |  |

**^*^** Amino acid sequences with under line indicate the restriction enzyme sites.
