# Supplementary material for: Molecular and Kinetic Properties of Two Acetylcholinesterases from the Western Honey Bee, Apis mellifera
Source: PLoS One. 2012 Nov 7;7(11):e48838. doi: 10.1371/journal.pone.0048838 (PMC3492254; doi:10.1371/journal.pone.0048838)
Supplement: Table S2 — IC50 (M) values of different inhibitors of recombinant AmAChE2 with or without pre-incubation with AmAChE1. (DOCX) [file pone.0048838.s006.docx]

**Table S2. IC_50_ (M) values of different inhibitors of recombinant AmAChE2 pre-incubated with or without AmAChE1^*^.**

| Inhibitor | Pre-BSA+AmAChE2 | Pre-AmAChE1+AmAChE2 | Ratio of IC_50_**^†^** |
| --- | --- | --- | --- |
| Chlorpyrifos oxon | (9.01 ± 2.57) × 10^-9^ | (2.83 ± 0.37) × 10^-8^ | 3.14 |
| Malaoxon | (9.98 ± 1.07) × 10^-7^ | (2.31 ± 0.50) × 10^-6^ | 2.32 |
|  |  |  |  |
| Carbofuran | (7.23 ± 0.15) × 10^-9^ | (3.27 ± 0.42) × 10^-8^ | 4.52 |
| Propoxur | (5.13 ± 0.08) × 10^-6^ | (1.98 ± 0.13) × 10^-5^ | 3.87 |

**^*^** Results are reported as the mean ± SD (n = 3).

**^†^** Pre-AmAce1+AmAChE2/ Pre-BSA+AmAChE2
